# Mechanically-Tunable Photonic Devices with On-Chip Integrated MEMS/NEMS Actuators

**DOI:** 10.3390/mi7040069

**Published:** 2016-04-16

**Authors:** Han Du, Fook Siong Chau, Guangya Zhou

**Affiliations:** Department of Mechanical Engineering, National University of Singapore, 9 Engineering Drive 1, Singapore 117575; du_han@u.nus.edu (H.D.); mpecfs@nus.edu.sg (F.S.C.)

**Keywords:** MEMS, NEMS, photonics, plasmonics, metamaterials, optomechanics

## Abstract

This article reviews mechanically-tunable photonic devices with on-chip integrated MEMS/NEMS actuators. With related reports mostly published within the last decade, this review focuses on the tuning mechanisms of various passive silicon photonic devices, including tunable waveguides, couplers, ring/disk resonators, and photonic crystal cavities, and their results are selectively elaborated upon and compared. Applications of the mechanisms are also discussed. Future development of mechanically-tunable photonics is considered and one possible approach is based on plasmonics, which can confine light energy in the nano-scale space. Optomechanics is another innovation, derived from the strong coupling of optical and mechanical degrees of freedom. State-of-the-art studies of mechanically-tunable plasmonics and on-chip optomechanics are also selectively reviewed.

## 1. Introduction

Semiconductor technology has grown rapidly in recent decades and nowadays, semiconductor devices are widely integrated with many modern equipment, from radios to satellites. However, the development of electronic devices has reached a bottleneck in terms of the operating speed due to factors such as parasitic capacitance. As a result, there is intensive research—both industrial and academic—on alternative and new technologies as alternatives to semiconductors [1]. One potential technology is photonics, which utilizes photons as the information carrier instead of electrons. Photonic signal processing has already been proven to possess ultra-high operation speed and low cross-talk between channels [2,3], although issues such as power consumption, cost and yield have hampered a wider industrialization of photonic technology [4]. The trade-off between electronics and photonics is widely discussed in the literature and there are suggestions that they can be merged together and complement each other [5,6,7,8,9,10]. 

Numerous studies have been carried out to develop methods to dynamically control photonic devices, *i.e.*, to realize tunable photonics. Current on-chip tuning mechanisms can be generally classified as thermo-optic [11], electro-optic [12,13] or mechanical methods. Among these, thermo-optic methods have the advantage of having a moderately large tuning range but have limited operating speed and high energy consumption [11]. Electro-optic methods are fast comparatively but they suffer from a minute tuning range because optical indices vary only slightly with modulation voltage [14]. Moreover, electro-optic methods—as well as thermo-optical methods—do not have the capability to drastically reconfigure photonic structures or significantly vary the strength of coupling between them. Despite this drawback, the relatively mature electro-optic methods currently dominate the field of tunable photonics while the nascent but promising mechanical methods are believed to be able to further accelerate the development of photonic technologies [15].

Mechanical tuning, *i.e.*, by mechanically moving or deforming structures, is a natural way to tune photonic devices and has many advantages over other methods such as a small footprint and compatibility of fabrication process with semiconductors. One widely-used group of mechanical tuning methods is based on micro/nano-electro-mechanical systems (MEMS/NEMS). The history of MEMS dates back to the 1960s and nowadays, MEMS devices are widely commercialized, e.g., MEMS actuators are utilized to drive the micro cameras on cell phones. With the development of micro/nano fabrication, the footprint of MEMS devices can shrink even further as more NEMS devices emerge. MEMS/NEMS devices benefit not only from a size advantage but they also have greatly enhanced performance. Many NEMS devices are currently being rapidly developed and some are employed for various state-of-the-art studies, e.g., nano-scale radiation heat transfer where ultra-precise and reversible mechanical control is required [16]. Since it is also relatively easy to integrate MEMS/NEMS actuators on-chip with functional components and combine their fabrication processes, it is reasonable to employ the technology to develop tunable photonic devices. 

## 2. Micro and Nano Fabrication

Standard micro fabrication processes have been established for released MEMS microstructures and they generally can be categorized as bulk micromachining or surface micromachining. Bulk micromachining forms structures by selectively etching inside the material substrate, in which case anisotropic etching is commonly used. Unlike bulk micromachining, surface micromachining builds structures by depositing and etching of different layers on top of the substrate. Bulk micromachined devices are space consuming and are usually limited to anisotropic materials. By comparison, surface micromachining permits flexibility in materials selection and enable high-resolution patterns and thus is widely used for the fabrication of MEMS/NEMS and photonic circuits. 

Traditional photolithography is a common method to define the patterns in micro fabrication and has a resolution in the micron- and submicron-scale. Improved methods, including electron beam lithography (EBL), focused ion beam (FIB), optical projection lithography, extreme UV lithography, X-ray lithography, *etc.* [17], are frequently used for nano patterning processes. Among them, EBL and FIB have an ultra-high resolution of several nanometers. No physical mask is required and patterns are formed by scanning a focused electron/ion beam dot by dot. Thus, they are flexible but time-consuming processes and are used extensively for fabricating nano-scale photonic structures such as photonic crystals and plasmonic nano dots. FIB even combines lithography and etching together to form the structures, while there is no etching involved in EBL. After defining the pattern, such as by EBL, material etching and deposition are utilized to form the structures. The most commonly used etching methods are ion milling [18] and reactive ion etching (RIE) [19]. Whilst ion milling is advantageous in the precise etching/patterning of materials like metals, it can cause crystal damage, induced by ion impact, when etching single crystal materials [20]. Conventional RIE usually cannot achieve high aspect ratios and near-vertical sidewalls. Thus, the Deep-RIE technique has been developed [21] and widely used in nano fabrication, especially of single crystal materials like single crystal silicon [22]. 

As for deposition, there are diverse techniques of chemical [23] and physical [24,25] vapor deposition and sputtering [26] of metallic and various kinds of dielectric materials. Last but not the least, chemical etching of sacrificial layers is always required to form suspended structures for mechanical tuning. Hydrofluoric (HF) acid etching of silicon dioxide (SiO_2_) is a common final fabrication process step to release and functionalize MEMS/NEMS devices. It is usually done using the HF vapor, instead of liquid, phase to avoid adhesion/stiction of nano structures arising from liquid surface tension [27].

## 3. MEMS/NEMS Tunable Si Photonics

Silicon photonic devices built with materials like single crystal silicon, poly-silicon, silicon oxide, silicon nitride, *etc.* are a well-developed group of on-chip photonic devices. They have already proven to have strong capability in infrared (IR) light confinement and modulation [28,29]. Silicon and silica are standard MEMS/NEMS materials as well and thus it is easy to on-chip monolithically integrate MEMS/NEMS actuators with photonic devices and combine their fabrication processes together. By mechanically moving or deforming photonic structures, diverse applications including tunable optical modulators, couplers, filters, routers, and switches are developed. 

In this section, the field of mechanically-tunable photonic devices with on-chip integrated MEMS/NEMS actuators is classified and reviewed based on the configuration of the photonic devices. 

### 3.1. Waveguide

**Figure 1:**
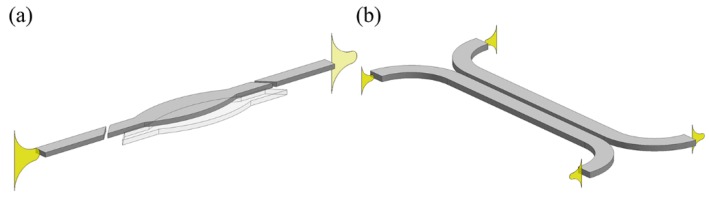
Schematics of (**a**) segmented waveguides and (**b**) coupled waveguides configuration.

A dielectric rectangular waveguide is a kind of conventional on-chip wave-guiding structure. The light is confined by total internal reflection when the index of the dielectric waveguide is higher than that of its surrounding. Mechanically-functional waveguides are generally based on configurations of segmented waveguides (see Figure 1a), coupled waveguides (see Figure 1b) and their derivatives.

**Figure 2:**
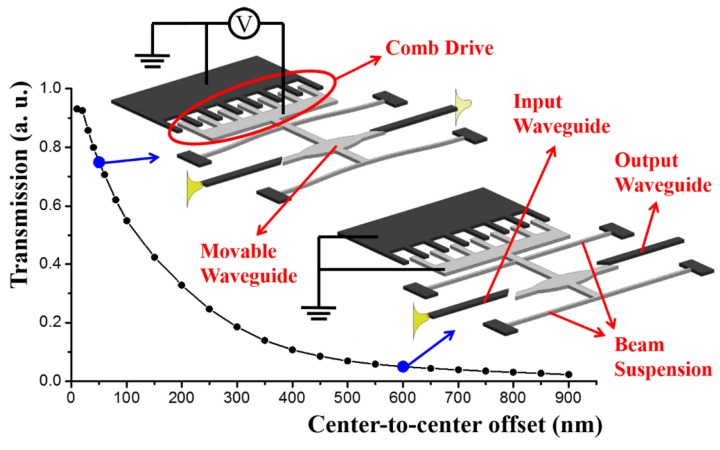
Finite difference time domain (FDTD) simulation results of the normalized transmission of the segmented waveguides configuration with respect to the center-to-center offset of the waveguide segments. The inserts denote the “on” and “off” states of the switch. The light grey parts denote movable structures while the dark grey parts indicate fixed structures. (The schematics in the figures below follow the same rule.)

Intuitively, it can be surmised that the transmission of light along segmented waveguides is roughly proportional to the total effective mode overlap integral of the waveguide cross-sections, which varies with the offset of the centers of the fixed and movable waveguide segments. The relationship between the normalized transmission (output power over input power) and the offset can be simulated using the finite difference time domain (FDTD) method. Typical results are plotted as the curve in Figure 2. The waveguide in the simulation model is of 400 nm width and 260 nm thickness, and the transverse-electric (TE) like mode of wavelength 1550 nm is excited. 

One way of building an optical waveguide switch is by mechanically moving the central movable waveguide segment [30], a configuration of which is illustrated by the inserts in Figure 2. As shown, the switch utilizes a monolithically integrated comb-drive actuator [31,32] that actuates based on electrostatic attraction and is one of the most widely-used MEMS/NEMS actuators. The movable fingers of the comb-drive are rigidly connected to the movable waveguide. The entire movable structure is supported by beam suspensions. At the initial condition, the movable waveguide has a large offset between the (fixed) input and output waveguides and the optical output is nearly zero, representing the “off” state of the switch. When there is a voltage across the fixed and movable fingers of the comb drive, the movable waveguide is displaced slightly and the offset reduced; the transmission thus increases, representing an “on” state. In this study, an extinction factor of about 15 dB was demonstrated between the “on” and “off” states at 1550 nm wavelength. The comb-drives and spring suspensions can follow standard designs [33] for large displacements with high positioning accuracy. This segmented waveguides configuration can also be applied to transduction [34] and displacement sensing [35] applications. Other optical switches and filters can also be achieved with a similar segmented waveguides design [36,37].

**Figure 3:**
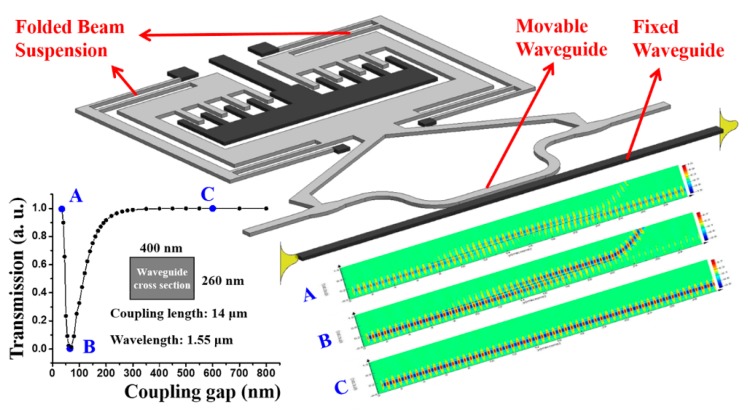
Schematic of the tunable waveguide coupler controlled by a comb drive actuator. FDTD simulated results of optical transmission and selected mode profiles are shown at left and right bottom.

The configuration illustrated in Figure 1b is known as a directional coupler, on which various functional devices can be realized. The transfer matrix obtained from spatial coupled mode theory (CMT) can be used to characterize such a device [38], *i.e.*,

(1) $$\left(\begin{array}{c} A(z)\\ B(z) \end{array}\right)=\left[\begin{array}{cc} e^{-j\delta z} & \\ & e^{j\delta z} \end{array}\right]\left[\begin{array}{cc} \cos(qz)+j\frac{\delta }{q}\sin(qz) & -j\frac{\kappa }{q}\sin(qz)\\ -j\frac{\kappa }{q}\sin(qz) & \cos(qz)-j\frac{\delta }{q}\sin(qz) \end{array}\right]\left(\begin{array}{c} A(0)\\ B(0) \end{array}\right)$$

where *A*(0) and *B*(0) are the modes launched into the coupler from two input ports while *A*(*z*) and *B*(*z*) are the modes emanating from two output ports, *z* denotes the coupling length, *δ* is the initial phase mismatch of the two input modes and $q=\sqrt{\kappa^{2}+\delta^{2}}$ with *κ* denoting the coupling coefficient between the two waveguides. Mechanically moving one of the dual coupled waveguides changes the coupling strength between them and thus varies the intensity and phase of the output light to realize optical modulation and switching. This mechanism can be applied to coupler switches [39,40,41,42,43,44], the configuration of which is illustrated in Figure 3. Folded beam suspensions instead of straight beam suspensions are used to support the movable structure in this case as they can release residual stresses arising from the fabrication process. Light is launched into and collected from the fixed waveguide. A comb-drive similar to that described above is used to control the coupling gap between the coupled waveguides by varying the applied voltage and thus modulating the output light. 

The curve at the bottom left of Figure 3 shows the FDTD simulation results of light transmission of such a waveguide coupler with dimensions and wavelength annotated in the figure. As can be seen, the coupling gap is tuned from 35 to 800 nm and the normalized transmission (output power over input power) roughly varies from 1 (at 35 nm marked by “A”) to 0 (at 70 nm marked by “B”) and back to 1 (at 600 nm marked by “C”). The optical mode profiles at these three critical positions are FDTD simulated and plotted at the right bottom of Figure 3. Generally, extinction ratio of over 10 dB at a wavelength of 1550 nm is experimentally demonstrated on the reported switches using the coupled waveguides configuration [40] with a measured rise time of about 18 μs [41]. In addition, a similar 1 × 3 switch has also been developed based on triple-coupled waveguides configuration [44]. 

**Figure 4:**
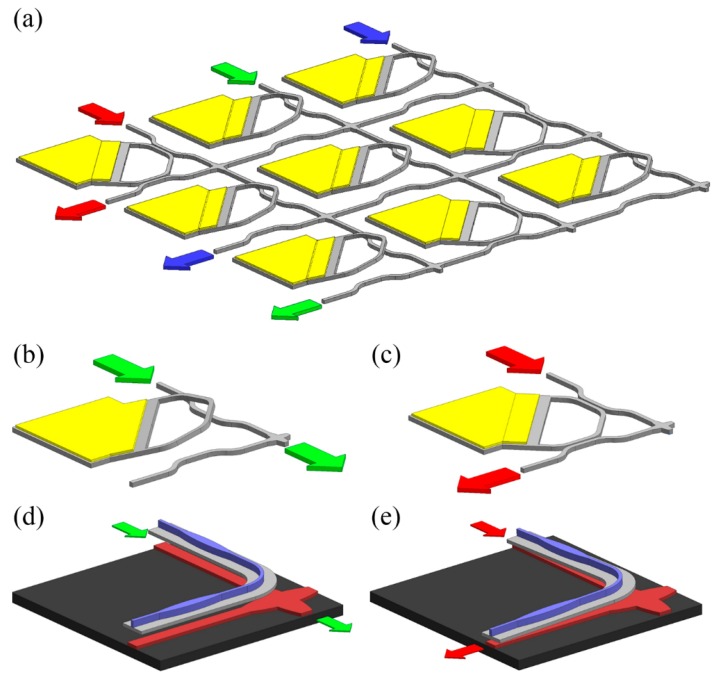
(**a**) Schematic of a large scale switch based on variable coupled waveguides. The yellow structures denote deposited gold layers. (**b**,**c**) Illustration of the through and drop states of a unit cell. (**d**,**e**) Illustration of the through and drop states of a unit cell of an improved design, in which the blue and red structures denote the suspended rib waveguide and the fixed rib waveguide, respectively.

Besides in-plane tuning of the dual-coupled waveguides, out-of-plane tuning is also investigated for on-chip large scale switches [45], the configuration of which is illustrated in Figure 4a. The through and drop states of a unit cell are given in Figure 4b,c, respectively. At the initial condition, the movable waveguide bends upwards (see Figure 4b) due to the residual stresses that are induced during gold layer deposition. In such a case, the light mainly launches out from the through port. With a voltage applied across the movable waveguide and the substrate, the waveguide will bend downwards (Figure 4c), in which case the light mainly launches out from the drop port. In this study, an on-chip 50 × 50 switch with extinction ration of 26 dB was realized. However, the insertion loss of the longest path was as high as 27.5 dB. As an improvement, a subsequent study in [46] utilizes vertically coupled wide rib waveguides, instead of narrow rectangular waveguides, to build a unit cell to reduce insertion loss and minimize device footprint. The vertical coupling gap is also electrostatically controlled. The through and drop state of a unit cell is illustrated in Figure 4d,e. An on-chip 64 × 64 switch with enhanced extinction ratio (over 60 dB), faster switching time (about 0.91 μs) and lower loss (about 3.7 dB) was realized.

Furthermore, not only can the output light intensities be controlled by tuning the coupling between the waveguides, but the output phase information can also be modulated. For example, a phase shifter has been developed based on such a principle [47], in which the electrostatic force is applied directly to the waveguides with a voltage across them. 

**Figure 5:**
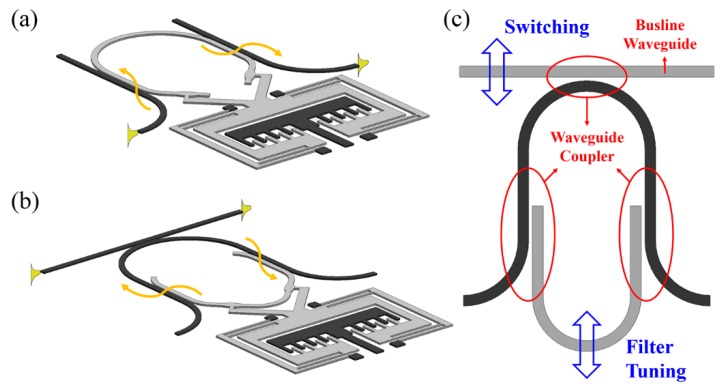
Schematic of (**a**) delay line (optical path length) tuning mechanism, (**b**) reconfigurable ring resonator consisting of two U-shaped waveguides, and (**c**) tuning of a mixed configuration (top view).

Besides changing the coupling gap, moving one of the waveguides along its length direction while maintaining a constant coupling gap will result in light modulation as a result of variation in the effective optical path length. This method is also known as delay line modulation and is used to control optical phase information [48]. As shown in Figure 5a, the phase modulator consists of two such waveguide couplers, where the free-standing movable waveguide is driven by a comb-drive actuator. Light is first coupled from a fixed input waveguide to the movable waveguide and then coupled back to the fixed output waveguide. A phase shift of 3π can be achieved with waveguide displacement of only 1 μm. Generally, such a design can be used in any photonic device, including tunable integrated interferometers that require large variations of optical path lengths. An excellent example demonstrating the use of such a tuning mechanism is a reconfigurable resonator illustrated schematically in Figure 5b [49,50]. As can be seen, the resonator consists of two U-shaped waveguides with two tunable waveguide couplers. Displacing the movable waveguide with the comb-drive changes the round-trip length of the ring resonator, thus altering its resonant wavelength. A relatively large tuning range can be obtained using such a mechanism. 

A combination of optical path length tuning and coupling strength tuning is also possible [51]. As shown in Figure 5c, a movable busline waveguide is coupled with a fixed U-shaped waveguide of a reconfigurable ring resonator. The busline waveguide and the movable U-shaped waveguide are controlled by individual comb-drives. Thus, the coupling gap between the busline waveguide and the fixed U-shaped waveguide, together with the round-trip path length of the ring resonator, can be tuned separately and thus the filter’s “on”-/”off”-state as well as its wavelength selectivity can be fully controlled. However, it should be noted that these reconfigurable ring resonators have low quality factors (*Q* factor).

### 3.2. Ring/Disk Resonator

Due to the intrinsic light field resonant enhancement, optical resonators are widely used for light control because they are wavelength-selective and usually have relatively high sensitivity to tuning. A well-known family of conventional micro optical resonators is micro ring/disk resonators, also known as whisper-gallery-mode (WGM) resonators. Reported ring/disk resonators fabricated with silicon/silica materials can have *Q* factors of over 10^8^ [52]. 

**Figure 6:**
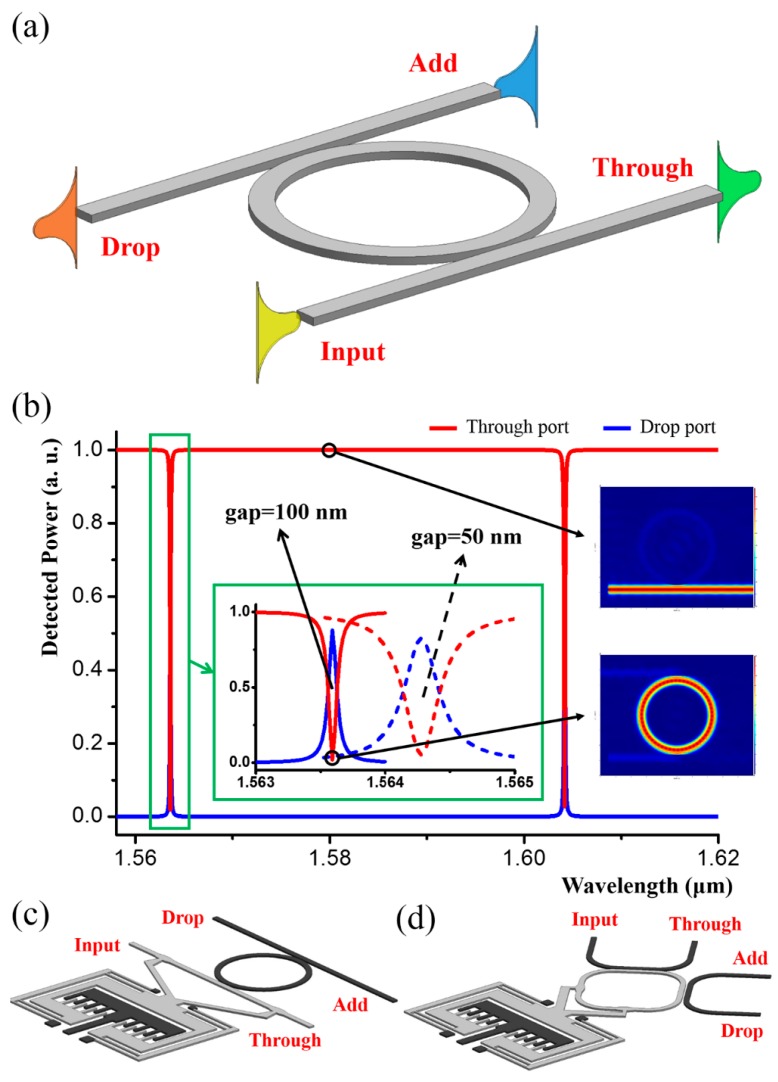
(**a**) Schematic of an add-drop filter based on a ring resonator. (**b**) FDTD simulation results of the filter. The red and blue curves denote the normalized power detected by the monitors at the through and drop ports respectively. Mode profiles of the through and drop state are given as the inserts on the right-hand side. (**c**,**d**) Schematics of the tuning mechanisms by mechanically moving (**c**) the channel waveguide and (**d**) the ring.

Generally, a ring resonator system consists of a ring and coupled channel waveguides, as illustrated schematically in Figure 6a, which is a classic wavelength-selective add-drop filter with four ports named as input, through, add and drop. FDTD simulation results of the filter is given in Figure 6b, in which the two curves denote the normalized power detected by the monitors located at the through and drop ports with respect to different wavelengths. As can be seen from the red curve (through port), there are two drops, denoting selective dropping wavelengths, which are the resonance wavelengths of the ring. The on-resonance mode profile is inserted in Figure 6b and it can be seen that light is mainly confined in the ring. However, when the input wavelength does not match the ring resonance, the transmission is nearly 100 percent. The mode profile in this case is also inserted in Figure 6b and it can be seen that nearly no light is coupled into the ring. A critical factor is the coupling coefficient between the waveguide and the ring and thus, tuning the coupling coefficient makes the filter adjustable, such as by changing the coupling gap between them. As illustrated in the inserts, as the gap shrinks to 50 nm, the dropping wavelength slightly red-shifts about 700 pm but the full width at half maximum (FWHM) broadens significantly. Such a tuning mechanism can be realized by translating the channel waveguide (see Figure 6c) [53,54,55] or the ring (see Figure 6d) [56] with the integrated comb-drive. The coupling gap between the ring and channel waveguides can be controlled by applying different voltages to the comb-drive.

**Figure 7:**
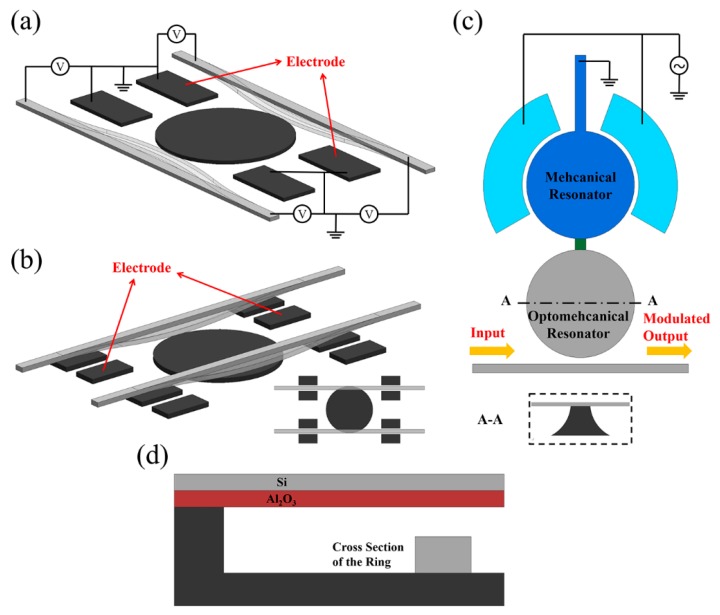
Schematics of (**a**) in-plane and (**b**) out-of-plane tuning mechanisms for dynamic add-drop filters, a disk resonator is coupled with suspended channel waveguides; (**c**) illustration of a dynamic optical intensity modulation controlled by an electrically-excited mechanical resonator (top view); and (**d**) cross-section view of the ring resonator system with thermal feedback using a bi-material thermal actuator.

A relatively simple way to tune the coupling strength between the channel waveguides and the resonator through deformation of the waveguides has also been reported [57]. As illustrated in Figure 7a, two free-standing waveguides are coupled with a disk resonator. When a voltage is applied across the waveguides and the on-chip fixed electrodes as shown in the figure, the flexible waveguides will bend towards the disk, thus obviating the need for an additional bulky MEMS actuator. The results show wavelength switching with an extinction ratio of 9 dB. 

Besides in-plane waveguides actuation, out-of-plane waveguides bending (Figure 7b) that achieved an enhanced extinction ratio of over 20 dB has also been reported [58,59]. As shown, the channel waveguides are suspended above the disk and the vertical coupling gap between them can be adjusted with a voltage applied across the suspended waveguides and the electrodes beneath them. When coupled out-of-plane, the waveguides are tangentially positioned as illustrated in the inserted top view to obtain a relatively strong coupling between the disk and waveguides. Such an add-drop filter has been demonstrated to have a wide tunable bandwidth, ranging from 12 to 27 GHz [60].

Besides static tuning, dynamic modulation based on such devices has also been demonstrated to have a high signal to noise ratio [61]. In the study cited in [62], dynamic optical intensity modulation controlled by an electrically-excited mechanical resonator is experimentally demonstrated. As shown in Figure 7c, the system mainly consists of a mechanical disk resonator (blue disks) that are excited electrostatically and an optomechanical disk resonator (grey disk) that is optically coupled with a channel waveguide. The A-A cross-sectional view is also inserted to show that the disk is supported at the center. The two disks are mechanically coupled with a short bridge (green bar in the figure), forming a two-degree of freedom mechanical oscillation system. The optical signal is launched into the channel waveguide and monitored at the output. The oscillation of the optomechanical disk resonator causes the coupling strength between the disk and waveguide to vary, resulting in dynamic signal modulation. The operational speed depends on the mechanical oscillation frequency of the disks; modulating rates of up to 235 MHz have been experimentally demonstrated [62].

The modes of actuation in the above-mentioned studies are all based on the electrostatic method. Another conventional MEMS driving method that is widely used is thermal actuation using bimorph materials [63]. Thermal actuation can also be used to tune photonic devices. An example is shown in Figure 7d in which the ring resonator is evanescently coupled to a bi-material cantilever consisting of Si and Al_2_O_3_ [64]. When the temperature is changed, the cantilever will bend due to induced thermal stresses, which in turn changes the coupling strength between the ring and the cantilever. This design was developed as a feedback mechanism to compensate for the thermo-optic effect in the system.

### 3.3. Photonic Crystal (PhC) Resonator

Photonic crystal [65,66], a kind of photonic bandgap structure, is an optically functional periodic structure. Analogous to semiconductor materials in which a periodic atom array can affect electron collective motions, photonic crystals can confine photons due to Bragg scattering, resulting in bandgaps. Photons with frequencies within photonic bandgaps cannot propagate in the crystal. Photonic components based on conventional rectangular waveguides can also be achieved using photonic crystals such as photonic crystal waveguides filters [28] and couplers [67]. However, one of the most attractive usages of photonic crystals is for building PhC cavities by inducing defects into them.

**Figure 8:**
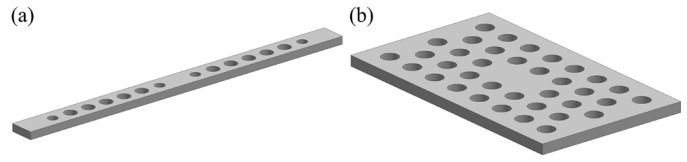
Schematics of (**a**) 1D PhC cavity and (**b**) 2D PhC cavity.

PhC cavities can be generally grouped into three types: one-dimensional (1D), two-dimensional (2D) and three-dimensional (3D) cavities. Of these, 3D cavities are difficult to fabricate on-chip and characterized, whilst 1D and 2D cavities are the most widely studied. Typical configurations are shown in Figure 8a,b. 2D PhC cavities are formed by inducing defects inside a planar photonic crystal [68]. Reported 2D cavities that are fabricated on silicon-on-insulator (SOI) material systems can have *Q* factors of over 10^6^ [69]. Similarly, 1D PhC cavities (also known as nanobeam cavities)—built by putting two PhC Bragg mirrors together to form a Fabry-Pérot-like resonator—can also have *Q* factors of over 10^6^ [70]. MEMS/NEMS actuators can also be integrated to vary the coupling between PhC cavities and waveguides, thereby enabling a range of tunable photonic devices. For example, Kanamori *et al*. developed a wavelength-selective channel drop switch with a 2D PhC in-plane coupled to a movable waveguide with the gap between them controlled by a comb-drive [71]. The drop efficiency can be controlled to 12.5 dB with a 600 nm gap variation. More interestingly, integration of MEMS/NEMS with PhC cavities can form mechanically-tunable photonic resonators/cavities, which are attractive for quantum signal processing [72], controlling of slowing and stopping light [73,74], tunable lasers [75] and many other applications [76,77].

**Figure 9:**
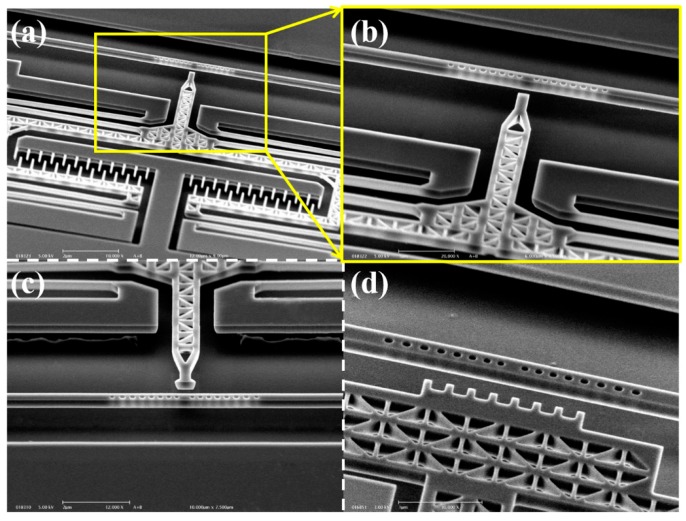
(**a**) SEM image of 1D PhC cavity resonance tuning mechanism using near-field perturbation approach with nano probe. Close-up views of (**b**) probe with a rectangular tip, (**c**) probe with a meniscus-like tip, and (**d**) probe with multiple tips.

Resonance control of optical resonators has long been studied and numerous mechanically tuning methods that do not modify the nature of the resonators have been developed. One of the more established methods utilizes evanescent field perturbation (or named as proximity perturbation), in which dielectric probes or cantilevers are suspended above 2D PhC cavities [78,79] or micro ring resonators [80,81] through complicated fabrication processes and driven by non-monolithic actuators. Besides the complicated system configuration, generally, only small tuning ranges can be achieved with relatively large mechanical displacements. To enhance the tuning range using evanescent field perturbation, Chew *et al*. studied near-field perturbation to 1D PhC cavities using nano-scale probes driven by monolithic NEMS comb-drive actuators, as shown in Figure 9 [82]. Using a probe having a rectangular tip of 400 nm width (see Figure 9a,b), a resonance shift of 1.1 nm is achieved when the tip to cavity gap changes from 750 to 100 nm, while a resonance shift of 2.25 nm is obtained with a probe having a meniscus-like tip with width of 800 nm (Figure 9c). For tuning with a rectangular tip probe, the *Q* factor and transmission of the cavity drops significantly, whereas only moderate degradation of the *Q* factor and transmission is observed with a meniscus-like tip probe. The near-field perturbation tuning method was later further enhanced with a nano-scale multi-tip probe (Figure 9d) and the results show a resonance wavelength variation of 5.4 nm with minimal *Q* factor degradation [83]. 

It should also be noted that the near-field perturbation mechanism can be applied to other photonic structures as well, e.g., waveguides [84] and waveguide couplers [85] for developing into optical phase modulators and switches. Besides light modulation, this mechanism is also widely used for transduction and sensing [86,87,88].

As mentioned earlier, the resonance tuning range that is obtained with near-field probe perturbation is generally not very large and moreover, additional energy losses that negatively affect the *Q* factor and transmission may result. To address these issues, the coupled-cavity tuning approach has been proposed and developed. When two cavities are coupled together, the resonance frequencies of their coupled supermodes can be adjusted by controlling the coupling coefficient between them, which is determined by the overlap integral of the mode fields of the two resonators. The coupling between two optical resonators could be described by the well-established temporal coupled mode theory (CMT) given below [89]:

(2) $$\begin{array}{l} \frac{d}{dt}a_{1}=j\omega_{0}a_{1}-j\mu a_{2}\\ \frac{d}{dt}a_{2}=j\omega_{0}a_{2}-j\mu a_{1} \end{array}$$

where *a_1_* and *a_2_* denote the optical modes on the two resonators, ω_0_ is the nature resonance frequency of individual resonators (considering two identical resonators) and μ denotes the coupling coefficient between the resonators. The resonance frequency of the coupled system can be given by

(3) $$\omega_{i}=\omega_{0}\pm \mu$$

**Figure 10:**
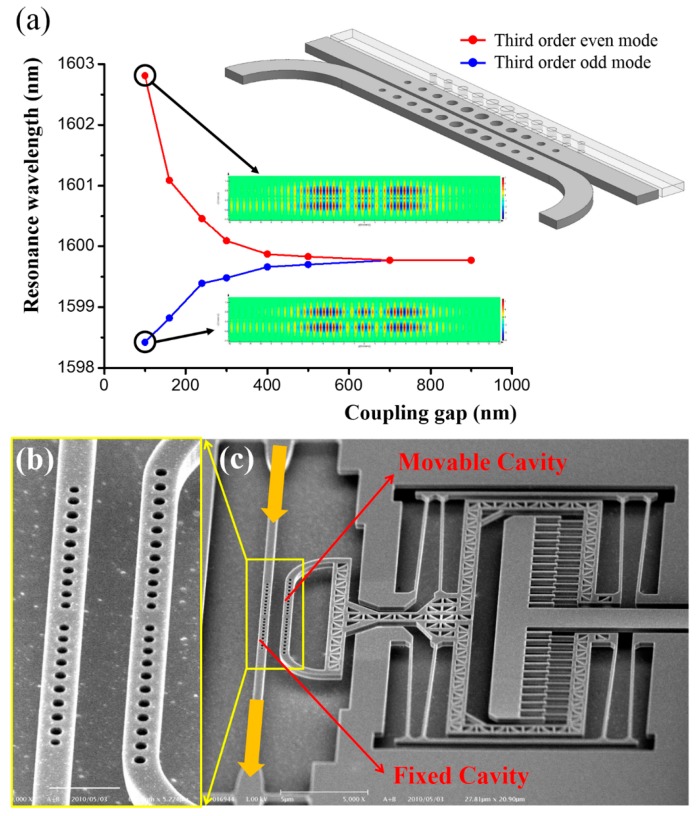
(**a**) FDTD simulation result of the third order even and odd modes indicating the wavelength shift of dual-coupled nanobeam cavities with respect to coupling gap variation. The mode profiles (when coupling gap is 100 nm) are inserted. (**b**,**c**) SEM images of (b) close-up view of the dual-coupled nanobeam cavities and (c) NEMS comb drive controlled dual-coupled nanobeam cavities system.

For nanobeam PhC cavities, the resonance with a lower frequency usually has a symmetric mode shape and is named as the even mode, while the resonance with a higher frequency possesses an anti-symmetric mode shape and is hence called the odd mode. Typical mode profiles of even and odd modes are shown as inserts in Figure 10a. As can be seen from Equation (3), resonant frequencies of the coupled cavities vary with coupling strength. Consequently, resonance control can be realized by tuning the coupling strength, which can be easily implemented with on-chip integrated MEMS/NEMS actuators. Taking a pair of 1D cavities in-plane coupled together as illustrated in Figure 10a as an example, the coupling strength between them can be modified by varying the coupling gap, which in turn will result in resonance shifts of the even and odd modes as shown in the FDTD-simulated curves in the figure.

A number of experimental demonstrations of the coupled-cavity resonance tuning approach have been reported. They are commonly based on 1D (nanobeam) PhC cavities due to their small footprint and ease of mechanical control. For example, when a voltage is applied across two in-plane-coupled 1D cavities, the induced electrostatic force pulls the two cavities together and thus changes the coupling strength between them [90]. A resonance wavelength shift of up to 9.5 nm on an even mode was observed. Using a similar method, vertically-coupled nanobeam cavities can also be electrostatically controlled [91]. In order to achieve large variations in coupling gap and, consequently, large wavelength tuning ranges, integration of MEMS/NEMS comb-drive actuators with PhC cavities is found to be highly advantageous.

Figure 10b,c show a dual-coupled nanobeam cavities system in which one of the coupled cavities is fixed and light is launched into and collected from this cavity. The other (movable) cavity is integrated with a NEMS comb-drive that controls the coupling gap. With this mechanism, up to 8 nm red-shift of an even mode and 3.8 nm blue-shift of an odd mode was observed in experiments without significant *Q* factor degradation when the coupling gap was changed from 750 to 150 nm [92]. To further broaden the resonance tuning range, a triple-coupled nanobeam cavities system was proposed and developed using two on-chip integrated comb-drives, where over 24 nm of resonance shift was experimentally demonstrated [93]. It is also interesting to note that in contrast to systems with large wavelength tuning ranges, ultra-precision resonance tuning over a small range has also been reported. For example, to accurately measure the opto-mechanical coupling coefficients of a set of dual-coupled 1D PhC nanobeam cavities, a comb-drive that is combined with a mechanical displacement shrinkage mechanism is used for ultra-fine control of the coupling gap and correspondingly, the resultant resonance [94]. 

**Figure 11:**
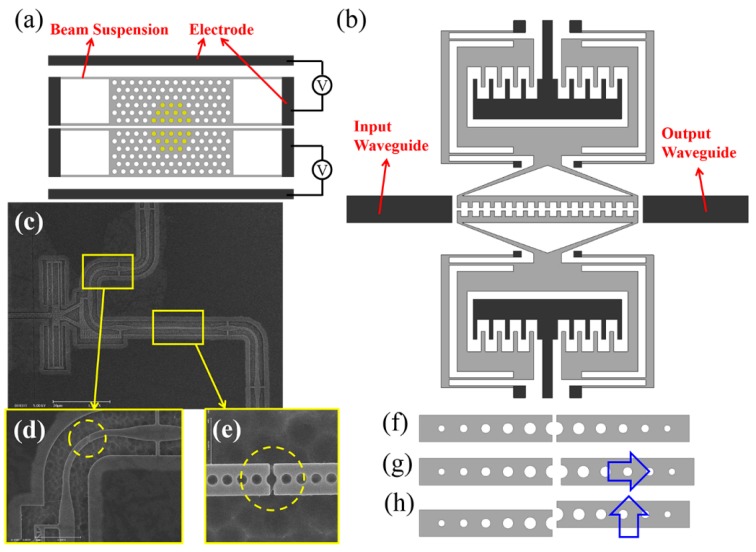
(**a**) Schematic of a tunable air-slot 2D PhC cavity with integrated electrostatic actuators. The yellow holes denote the defect region of the 2D PhC slab. (**b**) Schematic of a tunable transversely split nanobeam cavity. (**c**) SEM image of a tunable longitudinally split nanobeam cavity with close-up views given in (**d**) and (**e**). (**f**) Schematic of a longitudinally split nanobeam cavity with different tuning directions given in (**g**) and (**h**).

Another way to control the cavity resonance is by MEMS/NEMS-controlled reconfigurable or deformable PhC cavities. For 2D cavities, a design referred to as air-slot cavities, which have ultra-small mode volumes [95], is popular. As shown in Figure 11a, an air-slot is inserted in a planar photonic crystal slab and the dimensions of the air holes near the cavity center are adjusted to attain high *Q* factors. Due to dielectric discontinuity, the cavity mode is tightly confined and enhanced inside the narrow air gap between the two photonic bandgap structures. A mode volume of 0.14 of a cubic wavelength has been numerically demonstrated [96] and such cavities show promise in quantum electrodynamics studies. As can be seen from Figure 11a, the air gap can be easily tuned by means of an integrated electrostatic actuator. In the reported experiments, strong opto-electro-mechanical coupling and resonance shifting were demonstrated [97,98]. Besides air-slot 2D cavities, reconfigurable or deformable cavities can also be realized with 1D cavities. As shown in Figure 11b, the ladder-like cavity is transversely split by an air-slot into two symmetric parts, which are both movable and mechanically controlled by their individual comb-drives. The width of the air-slot is controlled by varying the voltage applied to the actuators. In the reported experiments, a resonance shift of up to 17 nm of the second-order mode was detected with an air-slot width increment of 26 nm while the *Q* factor varied only slightly [99].

1D PhC cavities can also be longitudinally split with an air-slot as shown in Figure 11f and thus made tunable. By changing the axial separation while keeping them aligned (Figure 11g), the *Q* factor of the cavity can be widely tuned and yet, the resonance wavelength is barely affected [100]. Figure 11c gives an SEM image of the device with close-up views of the disconnections shown circled in Figure 11d,e. This tuning mechanism was developed for applications in *Q*-switched lasers. Longitudinally-split nanobeam cavities can also be mechanically tuned by varying the offset laterally between the two components in-plane (Figure 11h) [101] and out-of-plane [102] while maintaining the same separation gap.

As discussed above, on-chip tuning mechanisms for photonic devices using monolithically integrated MEMS/NEMS generally have the merits of simple system configurations and fabrication processes, high precision, good reversibility, dynamic control capability, and low energy consumption. Therefore, the tuning methods have promising applications in future optical communication, sensing and quantum processing systems. With further advancements in nano fabrication techniques, dynamically tunable photonic devices with significantly reduced footprints yet have better performance and ultra-precise features are expected to evolve. Table 1 shows a comparison of MEMS/NEMS tunable Si photonic devices with different configurations, working principles, applications and remarks.

## 4. MEMS/NEMS Tunable Plasmonics and Chip-scale Optomechanics: An Outlook

In recent years, interest in photonics beyond dielectric materials has been growing rapidly [106]. For instance, a simple patterned metal layer can be engineered to possess much stronger light confinement compared with that of dielectric materials alone. Besides the innovation in materials, development of novel mechanical actuation methods is another attractive research direction. Among these, optical excitation could play an important role in future all-optical systems. In this section, MEMS/NEMS tunable plasmonic devices and on-chip integrated optomechanics are considered as possible candidates in an outlook of future development of mechanically tunable photonics.

### 4.1. MEMS/NEMS Tunable Plasmonic Devices

Plasmonics involving surface plasmons is a novel kind of photonics. Surface plasmons, a collective oscillation of electrons resonantly excited at the interface between a metallic and dielectric material, have been studied for years and are widely used for biomolecule detection [107,108,109]. Using surface plasmons, it is possible to circumvent the diffraction limit in dielectric materials, thus making it possible to localize electromagnetic waves in the nano-scale space. However, the intrinsic ohmic loss in metallic materials limits the wave- guiding capability of plasmonic structures. Though various diverse types of low-loss plasmonic waveguides, including hybrid waveguides, have been developed, there still remains a trade-off between mode size and propagation loss [106,110]. Compared with conventional wave-guiding structures using pure dielectric materials, light propagation in plasmonic structures still suffers much higher losses and encounters a narrow bandwidth limitation.

**Figure 12:**
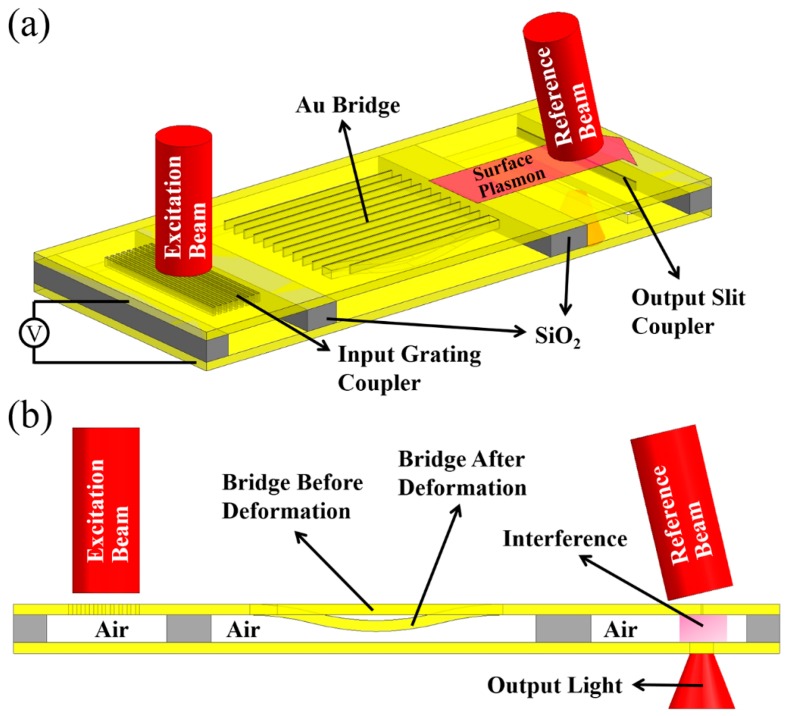
(**a**) 3D view and (**b**) cross-sectional view showing the schematic of the plasmonic phase modulator.

However, it is known that plasmonic devices can be embedded into photonic circuits as various kinds of photonic modulators [111,112,113]. The fabrication processes of such plasmonic devices are also well established [114]. Similar to MEMS/NEMS tuning of silicon photonic devices discussed in previous sections, on-chip mechanical tuning of plasmonic devices can be realized with electrostatic methods as well. For example, a phase modulator based on tunable metal-insulator-metal gap plasmons has been reported [115]. As shown in Figure 12, the device is fabricated on an Au-SiO_2_-Au stack and the SiO_2_ is finally removed. A free-space laser beam is focused on the input grating coupler and surface plasmons are excited at the gold-air interface. The surface plasmons propagate to the suspended Au bridge array where they are modulated. Finally, the surface plasmons are coupled to an output beam from the slit coupler and interfere with a reference beam. With this design, the phase information of the output light can be obtained. As shown in the figure, when a voltage is applied between the suspended gold bridges and the gold substrate, the bridges will bend downwards. Thus, the gap between the two gold layers where the surface plasmons are guided, shrinks. Since the phase velocity of the surface plasmons is extremely sensitive to the gap variations, a relatively large phase modulation can be realized with a small mechanical movement. The reported modulation depth is outstanding compared with convention Si photonic devices; the results show a phase shift of 1.5π achieved with only a 100 nm gap change. It should also be pointed out that there are numerous studies on gap guided surface plasmons in the literature [116,117].

**Figure 13:**
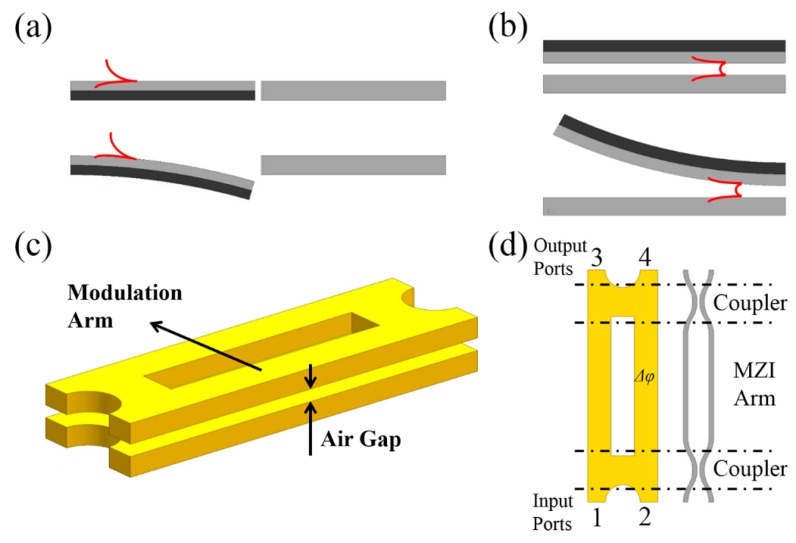
Schematic of the bi-material light (**a**) amplitude and (**b**) phase modulation mechanism. (**c**) Schematic of the plasmonic switch and (**d**) its equivalent optical circuit.

Other nanomechanical modulators based on plasmonic devices have been simulated and proposed (e.g., [118,119,120]), but have yet to be validated experimentally. In [118], a plasmonic light amplitude and phase modulator is proposed, which is temperature-controlled using bi-material thermal actuators. In Figure 13a,b, the dark grey structures denote silicon while the light grey structures are the silver layers. Variation of temperature will induce unequal thermal expansion of the two material layers and thus bending of the structure. Such a reconfigurable mechanism can be used to modulate light amplitude by inducing loss (Figure 13a) and modulate the phase by inducing phase delay (Figure 13b). The light amplitude and phase can be modulated simultaneously by cascading the two configurations. The phase modulation in the reported study is based on gap plasmons, like the phase modulator discussed in [115], and such a mechanism can be also applied to a plasmonic switch [119]. As illustrated in Figure 13c, the switch mainly consists of two vertically-stacked gold structures with an air-gap between them, where the gap plasmons are guided. By mechanically deforming the modulation arm in the vertical direction, the air gap changes, thus inducing a phase difference between the two arms of the Mach–Zehnder interferometer (MZI). This in turn varies the optical transmission at the two output ports (see the equivalent optical circuit in Figure 13d). To date, there are only a limited number of reports in this area and thus the authors feel that a wealth of opportunities exist in the development of mechanically tunable plasmonics in the future. 

**Figure 14:**
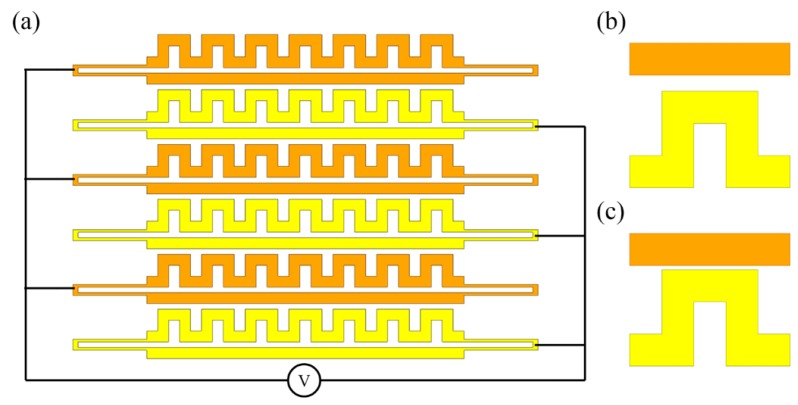
(**a**) Schematic of the electrostatically tunable plasmonic metamaterials. (**b**,**c**) The two states of a unit metamolecule.

In this review, the authors focus on light modulation in the guided mode. As for the field of free-space light modulation, metamaterials have been and are being extensively investigated. Plasmonic metamaterials [121,122] utilizing surface plasmon resonances to achieve extraordinary optical functionalities have been widely reported as well and one can find some studies on mechanically-tunable plasmonic metamaterials [123] among them. For example, as shown in Figure 14a, the gaps between strings and meanders in the constituent metamolecules can be controlled by the electrical configuration shown, thus forming an electrostatically-tunable plasmonic metamaterial [123]. Closed-up views of a single metamolecule before and after deformation are given in Figure 14b,c. Due to the ultra-small mass of the metamolecules and therefore, high mechanical resonance frequency, optical modulation up to the megahertz rate is demonstrable. Such mechanically-tunable metamaterials have been studied for years and numerous reports, including many review works [124,125,126,127], have been published. Consequently, a review of the study of mechanically tunable metamaterials will not be included here; however, some of these works have been included in Table 2 to compare the different kinds of photonic devices. 

### 4.2. Chip-Scale Optomechanics

**Figure 15:**
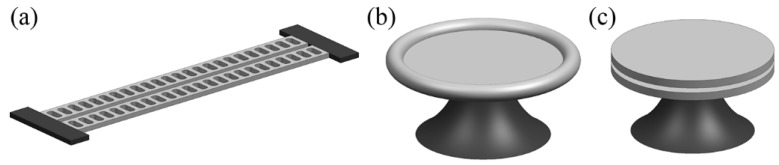
Schematic of (**a**) the coupled double clamped “zipper” cavities, (**b**) the toroid micro ring cavity and (**c**) the vertically stacked and coupled disk cavities.

For further development of mechanically controlled photonics, actuation using optical forces appears attractive due to its potential to be applied in all-optical systems. Driving photonic structures with optical force has already been experimentally proven [38,128,129] and some of its applications such as in filters and routers have also been demonstrated [130,131]. Being free from electrical parasitic couplings, mechanical oscillations driven by optical forces can reach ultra-high frequencies. Furthermore, on-chip integrated cavity optomechanics, derived from the coupling of optical and mechanical degrees of freedom, is a rapidly developing field that can bring about mechanical amplification or cooling as a result of the interaction between photons and phonons [132]. The experimental embodiments of chip-scale cavity optomechanics include coupled double-clamped “zipper” cavities (Figure 15a) [133], toroid micro ring cavities (Figure 15b) [134], vertically-stacked and coupled-disk cavities (Figure 15c) [135]. With the ability to measure mechanical oscillation beyond the standard quantum-limit [136,137], cavity optomechanics is currently being studied intensively for sensing applications with ultra-high resolution [138] and even used for investigation of gravitational waves [132,139]. It is believed that it holds great potential in both fundamental physics and innovative technologies for practical use.

A comparison of performance in light modulation of MEMS/NEMS tunable Si photonic devices, plasmonic devices, mechanically-tunable metamaterials, and optomechanic devices is presented in Table 2. The table includes many reported studies not discussed in the above text to complement and enrich this review paper. Unlike Si photonic devices, which are mostly tuned with electrostatic methods, mechanically-tunable metamaterials have diverse actuation methods and thus different actuation methods are also considered in the comparison.

## 5. Conclusions

In the present review, recent progress on mechanically-tunable photonic devices with on-chip integrated MEMS/NEMS actuators is reviewed based on publications mostly within the last ten years. Works are classified according to their device configurations. Typical and significant studies are discussed with their tuning mechanisms elaborated and results highlighted and compared. As an outlook of future development of mechanically-tunable photonics, state-of-the-art studies of MEMS/NEMS tunable plasmonics and chip-scale optomechanics are also briefly reviewed.

## Figures and Tables

**Table 1:** Comparison of different configurations of MEMS/NEMS tunable Si photonic devices.

	Metrics	Principle	Applications	Remarks	Reference
Configurations					
Waveguide	Segmented	Transmission modulation	Switch	High extinction ratio	[30]
			Motion detection	Moderate sensitivity	[35]
	Coupled	Evanescent coupling	Switch	High extinction ratio	[39,40,41,42]
			Phase modulator	High operation speed by deforming waveguides	[47]
			Wavelength tunable filter	Forming a ring resonator with adjustable round-trip length; low *Q* factor	[51]
	Evanescent field perturbation	Proximity perturbation	Phase modulator	High operation speed	[84]
			Switch	Moderate extinction ratio	[85]
			Switch, attenuator, or filter with non-monolithically integrated actuator	Complex fabrication process	[103,104]
Ring/Disk resonator	Add-drop filter with tunable gap between the resonator and channel waveguide	Coupling strength tuning; loss controlling	Switchable filter	Moderate extinction ratio	[53]
			Bandwidth tunable filter	Moderate bandwidth tuning range with slight resonance wavelength variation	[55,58,60,61]
			Switch	Moderate extinction ratio	[56]
	Add-drop filter with evanescent field perturbation	Proximity perturbation	Switchable filter	High extinction ratio	[80]
			Wavelength tunable filter	Small tuning range	[81,105]
PhC cavity	Coupled	Mode splitting variation by tuning coupling strength	Resonance control for such as tunable filter or router	Wide tuning range of resonance wavelength, with drawbacks of split modes	[90,91,92,93]
	2D air-slot cavity	Cavity deformation	Dynamic optical signal processing	High operation speed	[97,98]
	Transversely split 1D cavity	Cavity deformation	Resonance control	Wide tuning range of resonance wavelength	[99]
	Longitudinally split 1D cavity	Cavity deformation	*Q* factor tuning	Wide *Q* factor tuning range with slight resonance wavelength variation	[100]
	Evanescent field perturbation	Proximity perturbation	Resonance control	Moderate tuning range of resonance wavelength, yet with energy loss induced by perturbation	[78,79,82,83]

**Table 2:** Comparison of light modulation performances with MEMS/NEMS tunable Si photonic devices, plasmonic devices, mechanically tunable metamaterials, and chip-scale optomechanic devices.

	Metrics	Tuning Range	Modulation Depth	Operation Speed	Reference
Configurations & Actuation Methods					
MEMS/NEMS tunable Si photonic devices	Electrostatic	Moderate	High	Fast	Refer to Table 1
MEMS/NEMS tunable plasmonic devices, for guided mode	Electrostatic	Moderate	High	Fast	[115]
Mechanically tunable metamaterials, for free-space light	Thermal	Moderate	High	Slow	[140,141,142,143]
	Electrostatic	Wide	High	Moderate	[144,145,146,147,148,149,150,151,152,153,154,155,156,157,158,159,160,161]
	Magnetoelastic	Moderate	Low	Slow	[162,163]
Chip-scale optomechanics	Driving with optical force	Small	Low	Ultra-fast	[133,134,135]
